# Anoxic conditions are beneficial for abiotic diclofenac removal from water with manganese oxide (MnO_2_)

**DOI:** 10.1007/s11356-018-1569-2

**Published:** 2018-02-28

**Authors:** Wenbo Liu, Nora B. Sutton, Huub H. M. Rijnaarts, Alette A. M. Langenhoff

**Affiliations:** Sub-department of Environmental Technology, Wageningen University and Research, 6708 WG Wageningen, the Netherlands

**Keywords:** Manganese oxide, Abiotic pharmaceutical removal, Anoxic conditions, pH effects, MnO_2_ morphologies, MnO_2_ reactivity mechanism

## Abstract

This is the first study examining pharmaceutical removal under anoxic conditions with MnO_2_. This study compares the abiotic removal of seven pharmaceuticals with reactive MnO_2_ particles in the presence of oxygen (oxic conditions) and in the absence of oxygen (anoxic conditions). Due to the novelty of pharmaceutical removal under anoxic conditions, the influence of phosphate buffer, pH, and MnO_2_ morphologies is also examined. Results show that over 90% of diclofenac is removed under anoxic conditions. Additionally, we found that (1) anoxic conditions are beneficial for diclofenac removal with MnO_2_, (2) phosphate buffer affects the pharmaceutical removal efficiencies, (3) higher pharmaceutical removal is obtained at acidic pH compared to that at neutral or alkaline conditions, and (4) amorphous MnO_2_ removes pharmaceuticals better than crystalline MnO_2_. The pharmaceutical molecular structure and properties, MnO_2_ properties especially reactive sites of the MnO_2_ surface, are important for degradation kinetics. This study provides a fundamental basis towards understanding pharmaceutical degradation with MnO_2_ under anoxic conditions, and development of a cost-effective, sustainable technology for removal of pharmaceuticals from water.

**Electronic supplementary material:**

The online version of this article (10.1007/s11356-018-1569-2) contains supplementary material, which is available to authorized users.

## Introduction

Pharmaceuticals in the water cycle threaten the aquatic environment and drinking water resources. Already at low concentrations (ng/L~μg/L) (Simazaki et al. 2015; Ternes et al. 2015), pharmaceuticals can be toxic to aquatic organisms (Farré et al. 2008; Gilroy et al. 2014). As a result, pharmaceuticals discharged to water systems are seen as a priority concern of environmental regulators, and the European Union has added one of them, diclofenac, to the “Watchlist” (European Union 2013).

Removal of many pharmaceuticals such as carbamazepine, diclofenac, or metoprolol is poor in conventional wastewater treatment processes, such as activated sludge processes, due to the low biodegradability and limited sorption properties of many pharmaceuticals (Vieno and Sillanpaa 2014). Advanced technologies such as ozonation or photodegradation successfully remove selected pharmaceuticals from water and wastewater (He et al. 2016; Javier Benitez et al. 2009). However, these technologies require more energy inputs and operational costs, in addition to often high construction and maintenance costs, and produce intermediate compounds with unknown environmental effects.

A promising alternative method may be based on using manganese oxide (MnO_2_) to remove pharmaceuticals from water. MnO_2_, mainly referring to the oxide of manganese(IV) in previous studies, is also known as manganese dioxide (Chen et al. 2011; He et al. 2012; Huguet et al. 2013; Huguet et al. 2014). Using MnO_2_ can efficiently remove persistent pharmaceuticals like carbamazepine, and produce intermediates which are less toxic to the environment (He et al. 2012; Huguet et al. 2013). MnO_2_ is a common oxidant in soil, sediment, and marine environments, and these environments contain oxic (oxygen present) and/or anoxic (oxygen absent) zones (Kuan et al. 2013; Shin and Cheney 2004; Zhang et al. 2008). Most studies using MnO_2_ to remove pharmaceuticals are conducted under oxic conditions (Remucal and Ginder-Vogel 2014), because anoxic conditions have no effect or lower removal for pharmaceuticals. Oxygen can accelerate sulfamethazine oxidation by participation in the formation of intermediates (Gao et al. 2012), and for levofloxacin removal, rates under oxic and anoxic conditions are indifferent (Li et al. 2015).

Overall, these studies indicate that little is known about the abiotic removal of pharmaceuticals under anoxic conditions with MnO_2_. Further investigation under anoxic conditions might contribute to understanding how to improve the pharmaceutical removal with MnO_2_. From an application perspective, water treatment technologies commonly include oxic and anoxic steps. Investigating pharmaceutical removal under anoxic conditions with MnO_2_ may extend the application of this pharmaceutical removal technology. Additionally, applying anoxic conditions can reduce the construction and operation cost of maintaining oxic conditions in water treatment systems, which is an extra benefit using anoxic conditions. Furthermore, the effect of oxygen on pharmaceutical removal is inconsistent in different studies. Therefore, more studies are required to address pharmaceutical removal with MnO_2_ under both oxic and anoxic conditions, and to improve the understanding of the removal mechanisms.

Phosphate, pH, and MnO_2_ morphologies are known to affect the removal of organic compounds with MnO_2_ (Gao et al. 2012; Shin and Cheney 2004; Yao and Millero 1996). For example, various MnO_2_ morphologies have been tested to remove pharmaceuticals and other organic compounds, with amorphous MnO_2_ (birnessite) as most effective and most used (Remucal and Ginder-Vogel 2014). However, little is known about how these parameters affect the removal process under anoxic conditions.

In this study, a series of batch experiments with pharmaceuticals were conducted under oxic and anoxic conditions simulating the conditions encountered in nature as well as in wastewater treatment facilities. Seven widely used pharmaceuticals were selected and tested in the experiments. The effects of oxygen, phosphate, pH, and MnO_2_ morphologies were studied to better understand the removal processes involved and to optimize these towards the application of technology using reactive MnO_2_ for pharmaceutical removal.

## Materials and methods

### Chemicals

Caffeine, carbamazepine, diclofenac, metoprolol, naproxen, and propranolol were purchased from Sigma-Aldrich while ibuprofen was purchased from MP Biomedicals (detailed information in Table S1). Other chemicals were purchased from Sigma-Aldrich at 98% purity (for solids), or at HPLC or UPLC quality (for solvents). Pharmaceutical stocks were prepared with ultrapure water (18.2 MΩ cm, TOC = 18 ppb, Millipore, USA) and stored in amber glass bottles at − 20 °C. Other solutions were prepared with demineralized water (demiwater). Details are described in Text S1.

### MnO_2_ preparation

Amorphous MnO_2_ was obtained by freshly synthesizing prior to experiments as described (Langenhoff et al. 1997). Briefly, equal amounts of MnCl_2_ and KMnO_4_ were mixed, pH level was adjusted to ~ 10 with NaOH, and MnO_2_ was washed by centrifugation (Text S2). Amorphous MnO_2_ was used in all experiments unless specification. Crystalline MnO_2_ was purchased from Sigma-Aldrich (Fig. S1, S2).

### Batch experiments

One hundred twenty-five-milliliter glass bottles were filled with 50 mL MnO_2_ suspension (7 mM) in demiwater. Oxic experiments were prepared at atmospheric oxygen level. Experiments under anoxic conditions were prepared in the anaerobic glovebox with anoxic water and closed with a rubber stopper and aluminum cap before taking them out of the anaerobic glovebox. Outside the glovebox, the headspace was exchanged with 100% N_2_. All the experimental bottles were closed with rubber stoppers, crimped with aluminum caps, wrapped in aluminum foil to prevent photodegradation, and incubated without shaking at 30 °C.

Experiments were started by spiking bottles to achieve the final pharmaceutical concentration of 1 mg L^−1^. Aliquots were collected, and reactions were quenched immediately for analysis by centrifugation (10,000 rpm for 10 min). Blank experiments without MnO_2_ were prepared and conducted simultaneously with each batch of experiments. Sample collection and preparation before analysis are described in Text S3.

Experiments in 50 mM phosphate buffer with only diclofenac were conducted to compare the process under oxic and anoxic conditions. In addition, effects of pH and MnO_2_ morphologies under anoxic conditions were investigated with phosphate buffer solutions at pH 4~5 (4.5), pH 7.0, and pH 8~9 (8.5) (Text S1).

### Analysis

The pharmaceutical analysis was conducted as described previously using an ultra-performance liquid chromatography (UPLC, ultimate 3000, Thermo, USA) with a diode array detector (He et al. 2016). The pH level was determined by a pH meter (PHM210, MeterLab, Radiometer analytical). The Mn^2+^ analysis was conducted by an inductively coupled plasma spectrometer with optical emission spectroscopy (ICP-OES). MnO_2_ morphologies were characterized by X-ray diffraction. The MnO_2_ before and after the reaction with diclofenac and metoprolol was characterized via a Fourier-transform infra-red (FTIR, Bruker TENSOR 27) spectrometer. The figures of this study are analyzed and generated by Origin Pro 2015 and Microsoft PowerPoint 2007. Details are described in Text S3.

## Results and discussion

### Pharmaceutical removal under oxic versus anoxic conditions

**Fig. 1:**
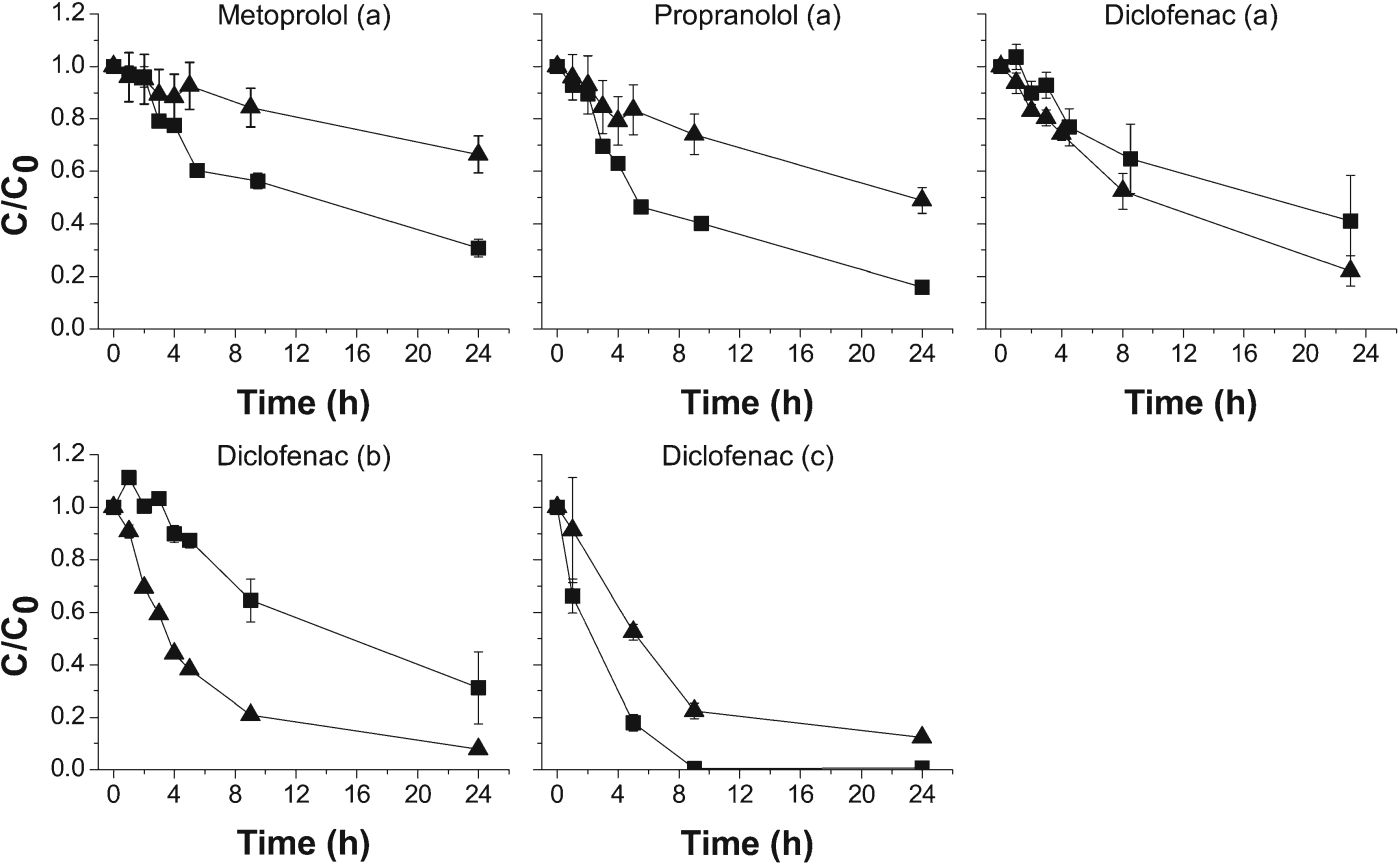
Pharmaceutical removal with MnO_2_ in demiwater with pharmaceutical mixture (**a**), demiwater with only diclofenac solution (**b**), phosphate buffer with only diclofenac solution under oxic conditions (black square) and anoxic conditions (black up-pointing triangle) (**c**). Experimental conditions: [MnO_2_]_0_ = 7 mM, [pharmaceutical]_0_ = 1 mg L^−1^, pH ~ 8.5. In phosphate buffer with diclofenac solution, [phosphate] = 50 mM, [ionic strength] = 0.1 M. Error bars are standard deviations determined

In the absence of MnO_2_, no removal is observed for all seven pharmaceuticals within 24 h under both oxic and anoxic conditions in all experiments (Table S3). In the presence of MnO_2_, metoprolol, propranolol, and diclofenac are removed within 24 h in both demiwater (Fig. 1a, b) and phosphate buffer (Fig. 1c), while no removal is observed for the other four pharmaceuticals (Fig. S3). Furthermore, the results show that removal efficiency of diclofenac is higher under anoxic conditions, while higher removal is observed under oxic conditions for metoprolol and propranolol. Diclofenac removal efficiencies of 78% under anoxic conditions and 59% under oxic conditions were observed after 24 h, incubating a solution of mixed pharmaceuticals in demineralized water (Fig. 1a). However, only 33% metoprolol was removed under anoxic conditions compared to 69% under oxic conditions. Similarly, 51% propranolol was removed under anoxic conditions compared to 84% under oxic conditions (Fig. 1a). Diclofenac removal efficiency in a mixture together with other six pharmaceuticals (Fig. 1a) was found to be lower than that in a demiwater system which only diclofenac was present (Fig. 1b). Under anoxic conditions, 92% diclofenac is removed with MnO_2_, while under oxic conditions, 69% diclofenac removal is observed (Fig. 1b).

In order to eliminate the effects of pH and ionic strength on pharmaceutical removal with MnO_2_ (Gao et al. 2012; Huguet et al. 2013), we control pH (~ 7) with 50 mM phosphate buffer and maintain the ionic strength (0.1 M) with NaCl. In further experiments with phosphate buffer, 90% of diclofenac is removed under anoxic conditions while nearly complete removal of diclofenac is observed under oxic conditions (Fig. 1c). The removal efficiency of diclofenac is similar under anoxic and oxic conditions. In previous studies, removal efficiency of organic matters including pharmaceuticals under anoxic conditions is either similar or lower than that under oxic conditions (Barrett and McBride 2005; Gao et al. 2012; Zhang and Huang 2005a). However, we notably observe that the removal efficiency of diclofenac under anoxic conditions can be higher than that under oxic conditions. This unique result directs our further studies on the mechanism of pharmaceutical removal under anoxic conditions with MnO_2_.

A pseudo-first-order model with an initial incubation period was applied to analyze the removal kinetics (Table 1), as performed in previous studies under oxic conditions (Jiang et al. 2010a; Zhang et al. 2008; Zhang and Huang 2005a). Comparison of the initial removal rate (*r*_obs, init_) and the initial removal rate constant (*k*_obs, init_) of different pharmaceuticals shows that oxygen affects pharmaceutical removal with MnO_2_. In demiwater with the pharmaceutical mixture and with only diclofenac, diclofenac removal is accelerated under anoxic conditions; metoprolol and propranolol removal rates are lower under anoxic conditions. Furthermore, diclofenac was removed at the highest rate when dissolved as a sole compound in oxic phosphate buffer containing MnO_2_.

**Table 1:** Initial removal rate (*r*_obs, init_, mg L^−1^ h^−1^, *R*^2^ = 0.80~0.97) and initial removal rate constant (*k*_obs, init_, h^−1^, *R*^2^ = 0.85~0.99) of pharmaceutical removal with MnO_2_ based on pseudo-first-order in first 5 h

Experimental solution	Matrix	pH	Compound(s)	*r*_obs, init_		*k*_obs, init_	
				(10^−2^ mg L^−1^ h^−1^)		(10^−2^ h^−1^)	
				Oxic	Anoxic	Oxic	Anoxic
Mixture of seven pharmaceutical	Demiwater	~ 8.5	Metoprolol	7.39	2.98^a^	9.21	3.18
			Propranolol	10.10	4.02	14.18	4.48
			Diclofenac	5.33	6.48	5.96	7.49
Only diclofenac present in solution	Demiwater	~ 8.5	Diclofenac^b^	4.70	9.06	5.56	18.13
Only diclofenac present in solution	50 mM phosphate buffer	~ 7.0	Diclofenac^b^	10.48	8.73	57.32	16.60

^a^Both *r*_obs, init_ and *k*_obs, init_ were calculated based on the periods 0–4 h

^b^Both *r*_obs, init_ and *k*_obs, init_ were calculated based on the periods 0–9 h

### Influence of pH and MnO_2_ morphologies on diclofenac removal

pH is an important parameter affecting pharmaceutical removal with MnO_2_. Previous studies show that MnO_2_ morphologies also influence pharmaceutical removal (Shin and Cheney 2004). However, our novel observation of diclofenac removal under anoxic conditions with MnO_2_ indicates that the removal mechanisms of pharmaceuticals with MnO_2_ under anoxic conditions might be different from removal mechanisms under oxic conditions. Therefore, it is important to investigate the effect of pH and MnO_2_ morphologies on diclofenac removal to understand the removal mechanism. We investigate the effect of pH and MnO_2_ morphologies using both amorphous MnO_2_ and crystalline MnO_2_ under anoxic conditions at pH ~ 4.5, pH ~ 7.0, and pH ~ 8.5 established with a 50 mM phosphate buffer.

Diclofenac removal efficiencies with MnO_2_ under anoxic conditions are inversely related to pH (Table 2). Within 48 h, diclofenac removal under anoxic conditions varies from 100% at around pH ~ 4.5 and pH ~ 7.0, to 70% at pH ~ 8.5 with amorphous MnO_2_. In contrast, diclofenac removal is notably lower with crystalline MnO_2_. Only 21% of diclofenac is removed with crystalline MnO_2_ at pH ~ 4.5. In the experiments carried out at pH ~ 7.0 and pH ~ 8.5, no diclofenac removal is observed with crystalline MnO_2_.

**Table 2:** Diclofenac removal efficiency under anoxic conditions at different pH conditions with two MnO_2_ morphologies after 48 h. Experimental conditions: [MnO_2_]_0_ = 7 mM, [diclofenac]_0_ = 1 mg L^−1^, [ionic strength] = 0.1 M

MnO_2_ morphologies	~ pH 4.5 (%)	~ pH 7.0 (%)	~ pH 8.5 (%)
Amorphous MnO_2_	100	100	71
Crystalline MnO_2_	21	0	0

### Discussion

Generally, removal of organic matters with MnO_2_ is a two-step process including adsorption and oxidation (Remucal and Ginder-Vogel 2014). The contribution of the two steps is various from different compounds (He et al. 2012; Xu et al. 2008; Zhang and Huang 2005b). Under oxic conditions, pharmaceutical removal can be accelerated by oxygen (Gao et al. 2012). However, this fails to explain why anoxic conditions are suitable for diclofenac removal in demiwater when oxygen is not present to participate in the removal process (Fig. S4). There are different intermediates formed under oxic and anoxic conditions during diclofenac removal with MnO_2_ (Fig. S4, S5). These intermediates have different adsorption affinities for the reactive sites on the MnO_2_ surface, which is possibly the key to explaining the differences between oxic and anoxic conditions. Based on the results, two factors appear to influence the efficiency of pharmaceutical removal and are elaborated below: (1) the pharmaceutical molecular structure and chemical properties, and (2) the MnO_2_ properties.

#### Pharmaceutical molecular structure and chemical properties

The molecular structure and chemical properties of pharmaceuticals are important in organic compound removal with MnO_2_. Previous studies show that oxidation with MnO_2_ in the presence of oxygen involves cleavage of the C–N bond of the organic compound. Metoprolol and propranolol have C–N bonds, in which the N atom is bound to an alkyl group. These compounds are similar to those tested in previous studies (Table S1, S2) in which oxic conditions promote the removal. This C–N bond cleavage can result in the formation of radicals in the presence of oxygen (Barrett and McBride 2005; Gao et al. 2012). Oxidation of diclofenac involves hydroxylation and decarboxylation instead of C–N cleavage (Huguet et al. 2013), which is a different mechanism than that of metoprolol and propranolol. This shows that the removal mechanism is closely related to the pharmaceutical molecular structure and chemical properties.

The pharmaceutical’s properties are also affected by pH. Due to the low pKa of diclofenac (pKa = 4.15), lower pH level results in a less negatively charged compound. This leads to less electrostatic repulsion between diclofenac and MnO_2_, which is also negatively charged (Murray 1974). It is speculated that lower pH level will lead to a higher affinity of diclofenac to adsorb onto the MnO_2_ surface and therefore has a more favorable first step in removal with MnO_2_.

#### MnO_2_ properties

The properties of MnO_2_ are also affected by pH. At acidic pH, MnO_2_ is also less negatively charged due to its isoelectric point, resulting in less electrostatic repulsion and better adsorption of organic compounds. In addition, the MnO_2_ redox potential increases from 0.76 V at pH 8.0 to 0.99 V at pH 4.0 (Lin et al. 2009). Thus, the degradation reaction is energetically more favorable at lower pH. Both factors may lead to faster degradation, as shown in our study (Table 2). This experiment uses neutral pH, which was found unfavorable for oxidation of pharmaceuticals in previous studies (Chen et al. 2011; He et al. 2012; Xu et al. 2008). In addition, there are less protons at the low redox potential of MnO_2_ at higher pH, which is crucial for the electron transfer from Mn(IV) to Mn(II). As a result, no removal of caffeine, carbamazepine, ibuprofen, and naproxen was observed in this study (Fig. S3), while the removal efficiency of metoprolol and propranolol is low under both oxic and anoxic conditions.

Different MnO_2_ morphologies have different properties affecting diclofenac removal. In our research, diclofenac removal is better with amorphous MnO_2_ than that with crystalline MnO_2_, which is in line with previously reported findings (Remucal and Ginder-Vogel 2014; Shin and Cheney 2004; Ukrainczyk and Mcbride 1992). Amorphous MnO_2_ particles are usually smaller than crystalline particles. Thus, the amorphous MnO_2_ particles have a larger surface area, which increases pharmaceutical removal. Unfortunately, due to the analytical limits, size analysis of amorphous MnO_2_ appeared technically not feasible (Fig. S6). In addition, amorphous MnO_2_ contains small amounts of Mn(III) which can increase MnO_2_ reactivity and oxidizing ability (Remucal and Ginder-Vogel 2014), thus promoting pharmaceutical removal even further.

In the presence of phosphate, diclofenac removal with MnO_2_ is slightly enhanced under oxic conditions than that under anoxic conditions. Using O_2_ to oxidize Mn(II) to Mn(III) is a thermodynamically favorable reaction. In the presence of phosphate buffer, phosphate can form Mn_3_(PO_4_)_2_ with Mn(II) from diclofenac oxidation (Eq. 1) (Jin et al. 2014).

1 $$ 3{\mathrm{Mn}}^{2+}+2{\mathrm{PO}}_4^{3-}\to {\mathrm{Mn}}_3{\left({\mathrm{PO}}_4\right)}_2 $$

**Fig. 2:**
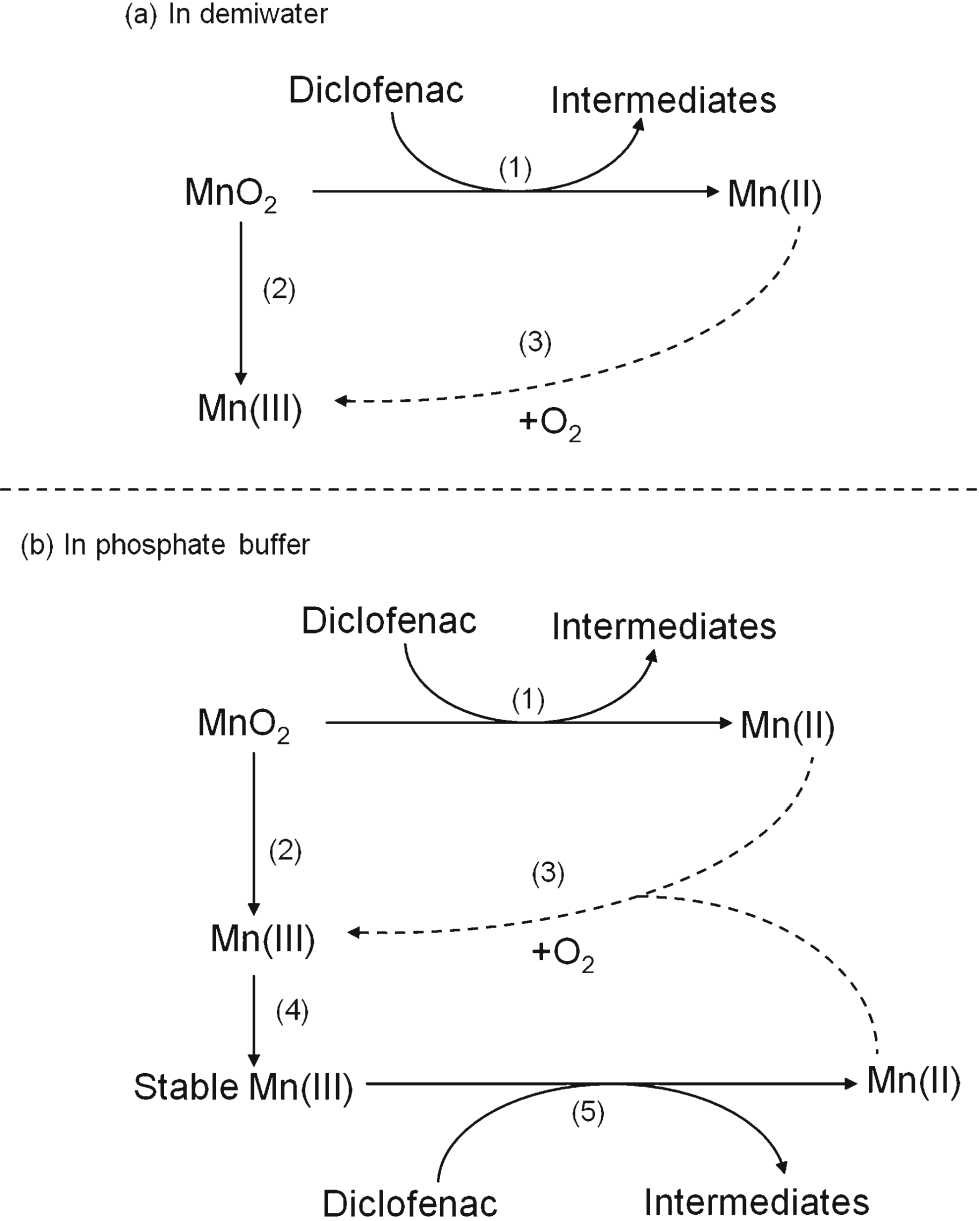
The effects of phosphate on diclofenac removal with MnO_2_ under oxic and anoxic conditions. Solid lines are processes under both oxic and anoxic conditions; dashed lines are the processes only under oxic conditions. (1) MnO_2_ removes diclofenac via oxidation and produces Mn(II) (Forrez et al. 2010; Huguet et al. 2013); (2) Mn(III) comes from MnO_2_ synthesis process (Remucal and Ginder-Vogel 2014); (3) Mn(II) is oxidized to Mn(III) by O_2_; (4) Mn(III) from MnO_2_ was stabilized by Mn_3_(PO_4_)_2_ formed via Eq. 1 (Jin et al. 2014); (5) Mn(III) oxidizes diclofenac and produces Mn(II)

Computations show that the chemical structure of Mn_3_(PO_4_)_2_ can stabilize Mn(III) and thereby facilitate Mn(II) oxidation to Mn(III) under oxic conditions (Jin et al. 2014). The Mn^2+^ analysis shows the presence of higher Mn(II) concentrations in phosphate buffer than in demiwater, which we explain as a result of larger amounts of Mn(III) formed under oxic conditions. Higher Mn(III) concentration is likely the reason that more diclofenac is removed than under anoxic conditions, as we observed (Fig. 1) and mechanistically present in Fig. 2.

#### Reactive sites on the MnO_2_ surface

The adsorption of organic molecules onto a reactive metal oxide surface is found to be the key parameter dictating removal of many organic compounds, and specifically to reactive sites on the MnO_2_ surface (He et al. 2012; Xu et al. 2008; Zhang and Huang 2005b). Our results with the mixed pharmaceutical solution in the demiwater suggest competition for reactive sites between diclofenac and the other different pharmaceuticals. This is evidenced by the lower diclofenac removal in the presence of other pharmaceuticals (Fig. 1a, b).

Based on our FTIR results, there was no obvious disappearance of reactive sites during diclofenac removal with MnO_2_ under both oxic and anoxic conditions (Fig. S5), possibly due to a relatively high concentration of MnO_2_ in the experiment. However, it is clear that the FTIR spectrums are different between the MnO_2_ before and after reacting with diclofenac, especially under anoxic conditions. This indicates that the intermediates from diclofenac change the MnO_2_ structure. This change may contribute to the better diclofenac removal with MnO_2_ under anoxic conditions.

In phosphate buffer, phosphate can reduce the diclofenac removal by being adsorbed onto the MnO_2_ surface and competing with DFC for the reactive sites of MnO_2_ (Yao and Millero 1996). Consequently, although the lower pH level in phosphate buffer should promote diclofenac removal (pH 7 in buffer versus pH 8~9 in demiwater), diclofenac removal is better in demiwater because MnO_2_ reactive sites are not blocked by phosphate (Table 1). However, similar removal efficiencies and kinetics in demiwater and phosphate buffer under anoxic conditions are observed (Fig. 1). This indicates there is a mechanism promoting diclofenac removal in phosphate buffer, which competes with the inhibition by phosphate adsorbing and occupying the reactive sites on the MnO_2_ surface. From previous studies, it is known that Mn(II) can occupy reactive sites on the MnO_2_ surface and then inhibit pharmaceutical removal (He et al. 2012; Xu et al. 2008). Our removal results in phosphate buffer show that 1.54 μM Mn^2+^ was generated under oxic conditions while 2.16 μM was generated under anoxic conditions. Less Mn(II) under oxic conditions resulted in possibly less formation of Mn_3_(PO_4_)_2_ via Eq. 1, which presumably led to more available reactive sites for diclofenac removal. Under anoxic conditions, the balance of these promoting and inhibiting effects by adsorbing phosphate leads to similar diclofenac removal in demiwater and phosphate buffer.

## Conclusions

In conclusion, this study addresses the knowledge gap surrounding pharmaceutical removal under anoxic conditions (absence of oxygen) with MnO_2_. Results show that anoxic conditions are beneficial for diclofenac removal with MnO_2_. In demiwater, anoxic conditions show higher diclofenac removal compared to oxic conditions. In phosphate buffer, anoxic conditions resulted in similar diclofenac removal (10% difference) comparing to oxic conditions. Both pH and MnO_2_ morphologies influence the removal process and its efficiency. Since both demiwater and phosphate buffer suggest that anoxic conditions are as good as, or even better than, oxic conditions in diclofenac removal from water with MnO_2_, the less potential cost in processes under anoxic conditions is more attractive and promising in treating water and wastewater containing pharmaceuticals. The results show that amorphous MnO_2_ is the most suitable material for further research and application, and the most optimal and applicable conditions are at neutral pH in anoxic systems. By using a more favorable pH (acidic pH), the removal of all the pharmaceuticals can be expected under anoxic conditions. To our knowledge, this is the first study discussing pharmaceutical removal with MnO_2_ under anoxic conditions. Using anoxic conditions is less energy-consuming compared to using oxic conditions (aeration), and Mn can be regenerated and recycled via a biological or chemical process (Jiang et al. 2010b; Liu et al., Biological regeneration of manganese (IV) and iron (III) for anaerobic metal oxide-mediated removal of pharmaceuticals from water, submitted; Tebo et al. 2004). Overall, this study contributes to (1) understanding pharmaceutical removal in the absence of oxygen, (2) improving the knowledge of pharmaceutical removal mechanisms with MnO_2_, and (3) providing fundamental insight into a MnO_2_-based process which may lead to a more sustainable technology for pharmaceutical removal.

## Electronic supplementary material

ESM 1 (PDF 1060 kb)
