# Supplementary material for: Anoxic conditions are beneficial for abiotic diclofenac removal from water with manganese oxide (MnO2)
Source: Environ Sci Pollut Res Int. 2018 Feb 28;25(10):10141–7. doi: 10.1007/s11356-018-1569-2 (PMC5891569; doi:10.1007/s11356-018-1569-2)
Supplement: Supplementary file 1 — (PDF 1060 kb) [file 11356_2018_1569_MOESM1_ESM.pdf]

Supplementary materials

Article title: Anoxic conditions promote abiotic diclofenac removal with manganese oxides

Journal: Environmental Science and Pollution Research

Author(s): Wenbo Liu, Nora B. Sutton, Huub H. M. Rijnaarts, and Alette A. M. Langenhoff*

***Corresponding Author**

Dr. Alette A M Langenhoff

alette.langenhoff@wur.nl

Sub-department of Environmental Technology, Wageningen University and Research, 6708 WG, Wageningen, the Netherlands

TEXT S1. Solution preparation

Anoxic water. The anoxic water was prepared by boiling the demineralised water for 5 minutes. Then the boiled water was transferred to a glass bottle and bubbled with N_2 until it had cooled down to room temperature. The water was then sealed and stored at room temperature.

Pharmaceutical stock. The pharmaceutical stock was included pharmaceutical mixture stock and diclofenac stock. The pharmaceutical mixture stock was prepared by dissolving 20 mg of every seven pharmaceuticals with 1 L ultrapure water. The final concentration of each pharmaceutical in pharmaceutical mixture stock is about 20mg/L. The diclofenac stock was prepared by dissolving 125 mg diclofenac with 50 mL ultrapure water. The final diclofenac concentration in diclofenac stock is about 2500 mg/L.

Reaction solution. The reaction solution contained 7 mM MnO_2, 50 mM buffer to maintain constant pH at 7, and an appropriate amount of NaCl to maintain constant ionic strength at 0.1 M. The buffer is H_3PO_4/NaH_2PO_4 for pH ~ 4.5, NaH_2PO_4/K_2HPO_4 for pH~7.0, and K_2HPO_4 for pH ~8.5. The reaction solution was prepared with both normal demineralised water and anoxic water.

TEXT S2. MnO_2 generation

Both 0.4 mM $MnCl_2$ and 0.4 mM $KMnO_4$ were prepared with demineralised water. While stirring the $KMnO_4$ solution continuously, equal volume of $MnCl_2$ was added. Then, NaOH (1N) was added into the mixture to bring the pH to 10. The MnO_2 solid then centrifuged at 5000 rpm for 15 min and then resuspended by anoxic water. The centrifigation and suspended in anoxic water were repeated for six times. Suspension of MnO_2 in anoxic water were stored at 4°C for the addition to batch experiment.

TEXT S3. Analysis

Pharmaceutical analysis.1 mL samples were collected and centrifuged at 10000 rpm for 10 min. The supernatant then transferred to amber vials. 50 µL of internal standard (5mg L^{-1} fenoprofen) was added into the sample. The samples were stored at -20 ˚C before analysis.

The pharmaceutical analysis was performed as described [1] by a ultra-performance liquid chromatography (UPLC, ultimate 3000, Thermo, USA) with a diode array detector, and a CSH phenyl-Hexyl column (1.7µm, 130 Å, 2.1 × 150 mm). A mixture of water with 0.1% formic acid (solution A) and acetonitrile with 0.1% formic acid (solution B) was used as the mobile phase. The analysis started with 100% solution A for 0.5 min. Then it decreased to 20% at 13 min. After staying for 3 min, it went back to 100% at 17 min and stopped at 22.4 min. The flow rate is 0.3mL/min while the oven is at 40 °C. The injection volume of samples was

10 μl. The pharmaceutical concentration was calculated based on relative area (area of pharmaceutical/area of internal standard) and the slope of the calibration curve.

Mn^{2+} analysis. The Mn^{2+} generated during the removal processes was determined by an inductively coupled plasma spectrometer with optical emission spectroscopy (ICP-OES, Vista MPX Simultaneous, Varian Inc. (Part A), USA). Controls with only MnO_2 and buffers but without pharmaceuticals showed no Mn^{2+} produced.

MnO_2 morphologies analysis. The two kinds of MnO_2 were characterized by X-ray diffraction (XRD, D2 PHASER, Bruker, Germany). The results showed in Figure S1. In addition, microscopes were also used to characterized the MnO_2 morphologies (Figure S2)

Fournier-Transform Infra-Red spectrometers. The MnO_2 solid with diclofenac or metoprolol as described in previously (section 2.3) were collected by centrifugation (5000rpm, 15 min) after 24h under both oxic and anoxic conditions in deminerized water. Control sample, bare MnO2 without pharmaceuticals, was also collected based on the same method. The samples were freeze-dried before analysis. All the samples were analyzed by a Fournier-Transform Infra-Red spectrometers (Bruker TENSOR 27). The reference spectrum of untreated diclofenac and metoprolol standard was also acquired in the same analytical condition.

TABLE S1. Structure and properties of selected pharmaceuticals in this study

Pharmaceutical[a]	CAS No.	Chemical Structure	pK_a	$LogK_{ow}$
diclofenac (DFC)	15307-79-6	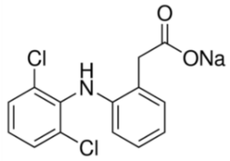	4.15	4.57
Caffeine (CAF)	58-08-2	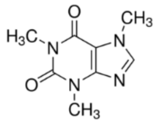	10.4 (at 40 ℃)	-0.07
Carbamazepine (CBZ)	298-46-4	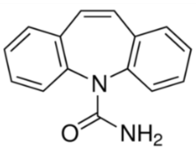	13.9	2.45
Ibuprofen (IBP)	15687-27-1	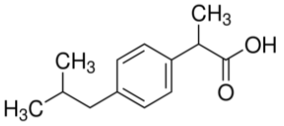	4.91	3.97
Metoprolol (MET)	56392-17-7	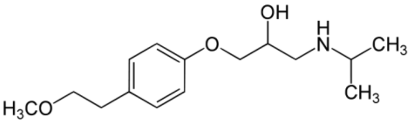	9.5	1.88
Naproxen (NAP)	22204-53-1	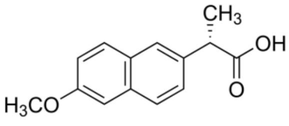	4.15	3.18
Propranolol (PROP)	318-98-9	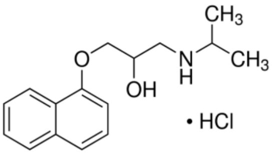	9.42	3.48

[a]Text in the brackets is the abbreviation of each pharmaceutical.

TABLE S2. Structure and properties of glyphosate and sulfamethazine

Compound	CAS No.	Chemical Structure	pK_a	$LogK_{ow}$
glyphosate	1071-83-6	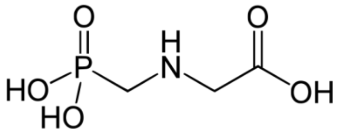	0.8 (1st phosphonic)	-2.8
sulfamethazine	57-68-1	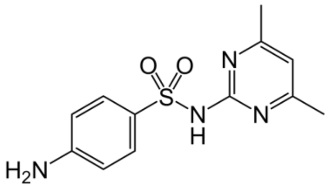	7.59	0.89

TABLE S3. Pharmaceutical concentration (C/C_0 at t= 24 h) in absence of MnO_2 in demineralised water. Experimental conditions: $[pharmaceutical]_0 = 1 mg \cdot L^{-1}$, pH ~8.5.

	caffeine	carbamazepine	diclofenac	ibuprofen	metoprolol	naproxen	propranolol
Oxic conditions	1.02	1.01	1.00	1.03	1.01	1.02	1.01
Anoxic conditions	1.02	1.01	1.09	1.04	1.01	1.02	1.02

TEXT S4. Characteristics of two MnO_2 morphologies

X-ray diffraction spectrum and microscopy clearly showed the differences between two MnO_2 (Figure S1, S2). The MnO_2 prepared in this experiments is amorphous while the MnO2 purchased commercially (from Sigma-Aldrich) is crystalline MnO_2.

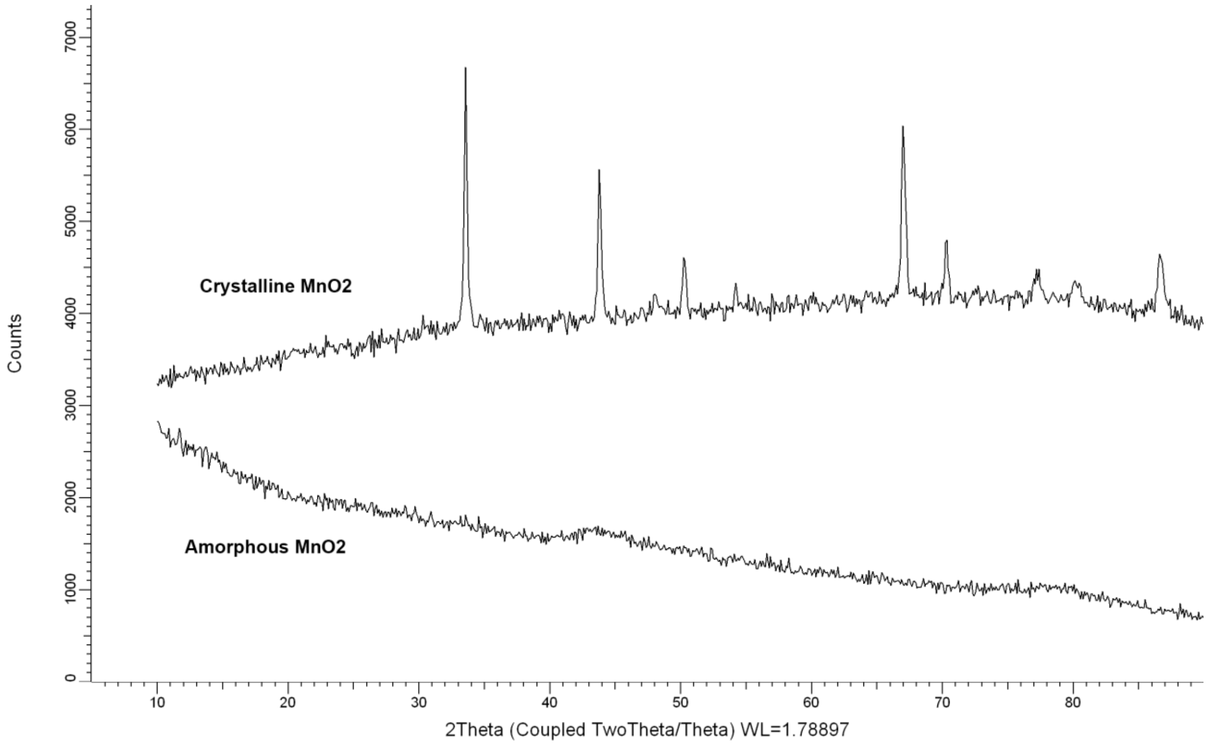

FIG. S1. X-ray diffraction spectrum of amorphous MnO_2 and crystalline MnO_2

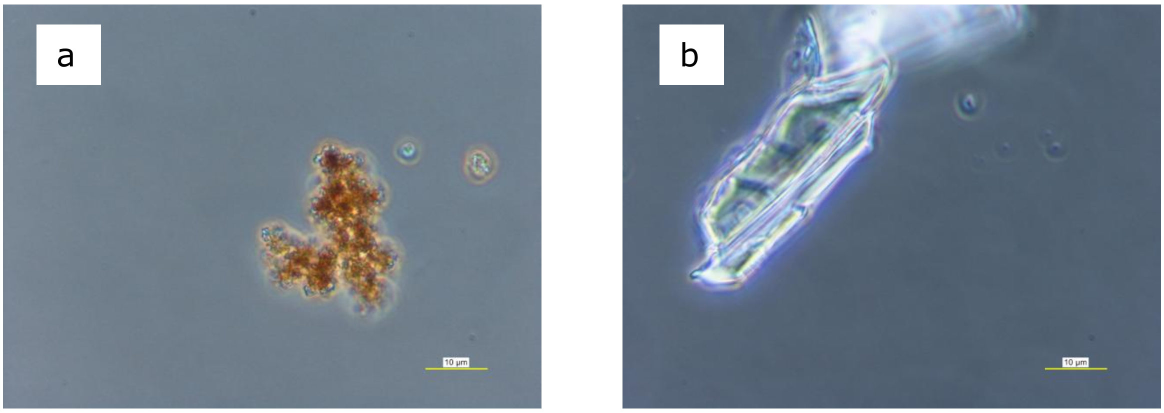

FIG. S2. Microscopy of (a) amorphous MnO_2 and (b) crystalline MnO_2 at 100 times magnificence.

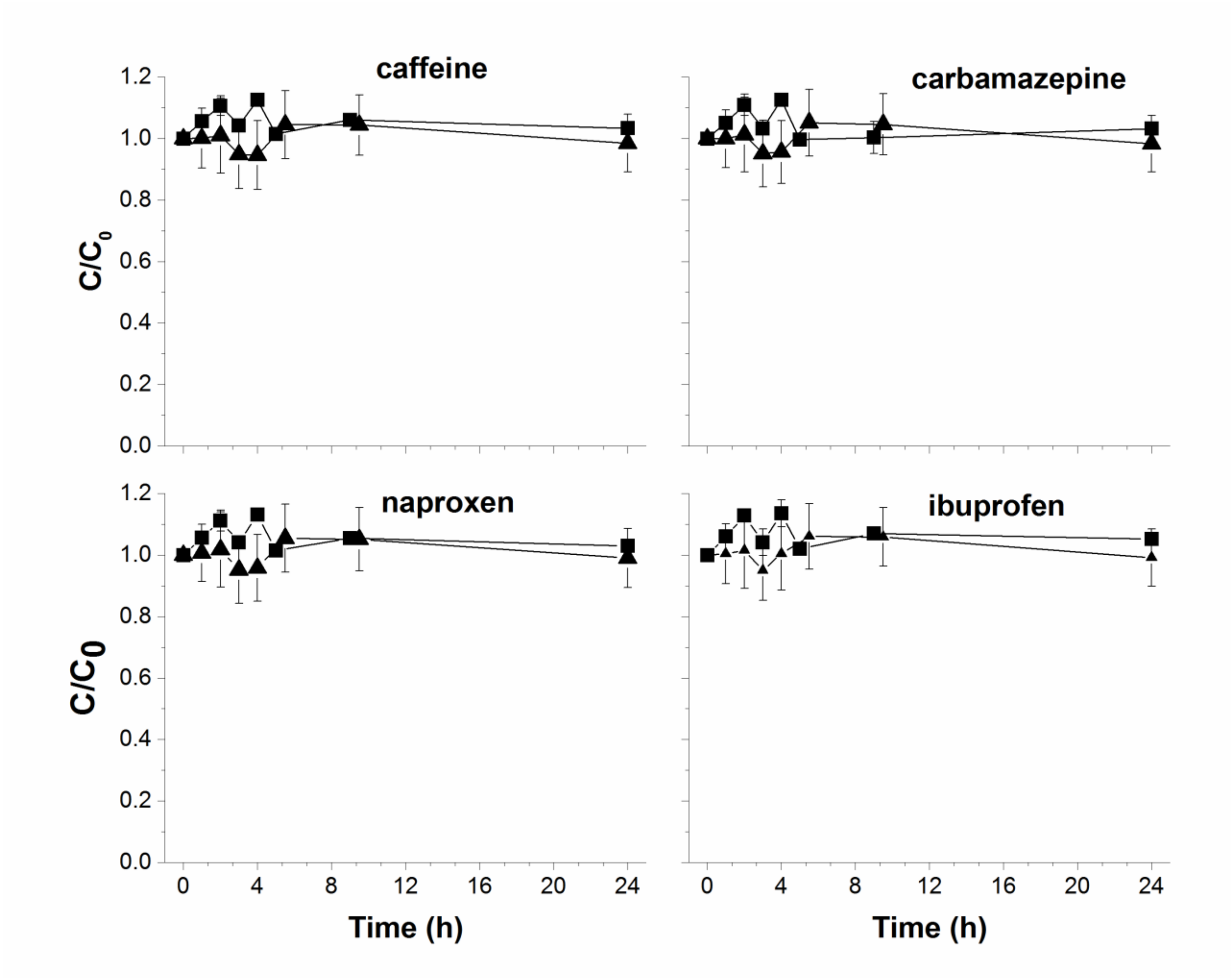

FIG. S3. Pharmaceutical removal with MnO_2 in demineralised water with pharmaceutical mixture under oxic conditions (■) and anoxic conditions (▲). Experimental conditions: $[MnO_2]_0$=7 mM, $[pharmaceutical]_0$=1mg·L^{-1}, pH ~8.5. Error bars are standard deviations.

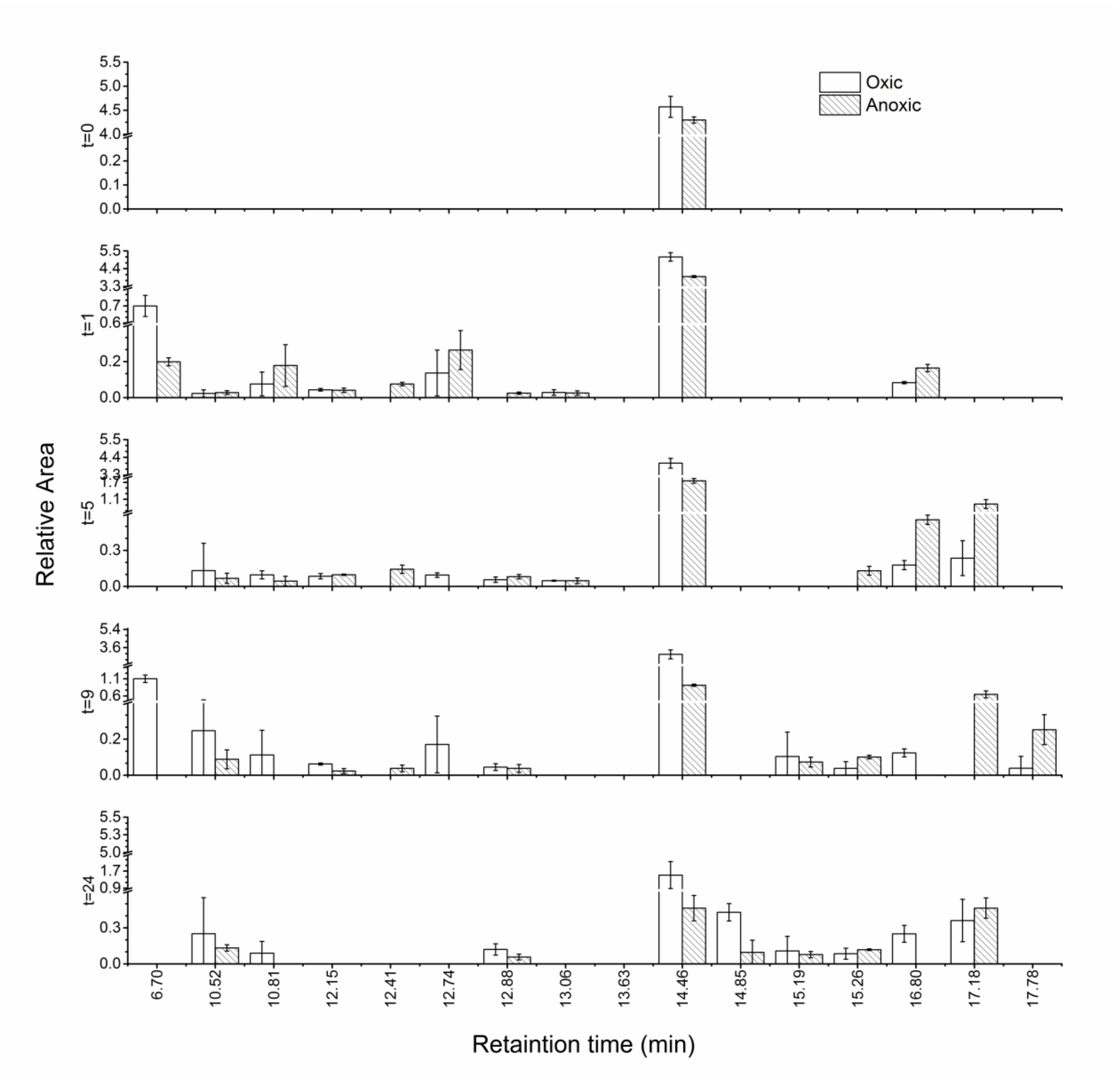

FIG. S4. Presence and variation of intermediates from pharmaceuticals under oxic and anoxic conditions at different reaction time point showed in chromatography spectrum in demineralised water system. The relative area was the ratio between the area of the potential intermediates and the area of internal standard (fenoprofen). Error bars are standard deviations

In the chromatography spectrum, the peaks at different retention time (RT) are representative different intermediates of diclofenac. The diclofenac is observed at RT=14.64 min. Due to the practical limits, it is impossible for us to identify the formula or structure of intermediates in diclofenac removal with MnO_2. However, the chromatography clearly shows that the intermediates formed under oxic and anoxic conditions are different. This may indicate that oxygen contributes to forming the intermediates in our experiments, which is observed previously .

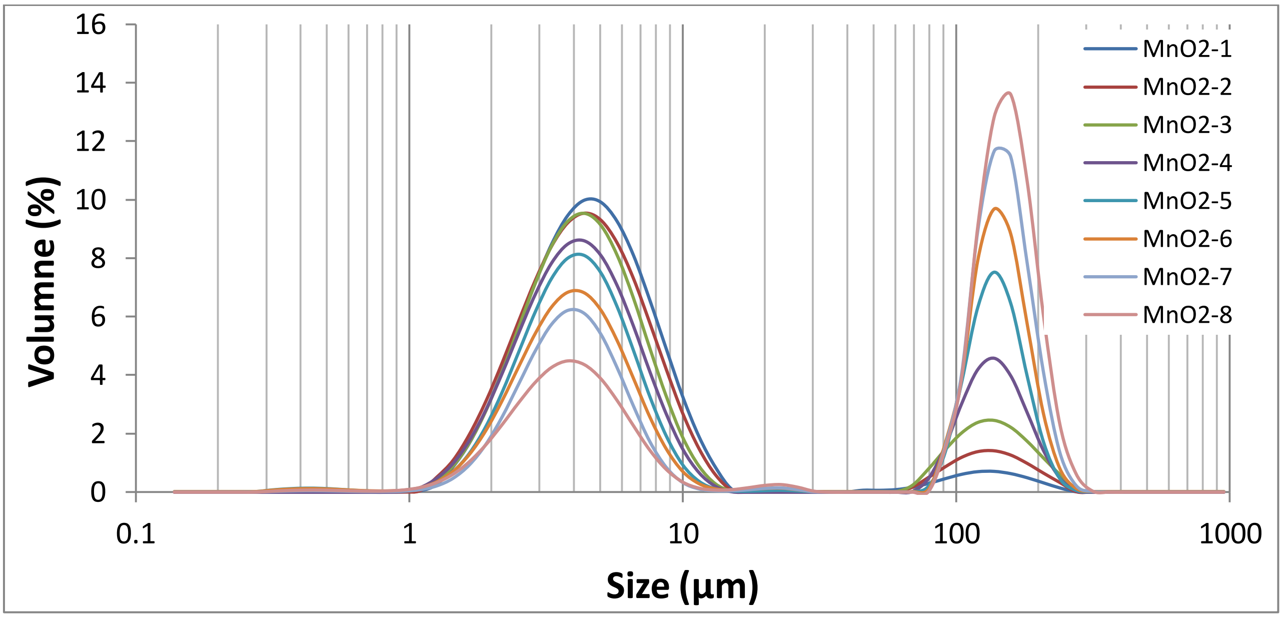

FIG. S5. Size analysis of amorphous MnO_2 (8 times analysis for the same sample, 40 sec/sample).

Size analysis was performed by a laser size analyzer (Mastersizer 2000, Malven, UK). The size of MnO_2 particles keep increasing during the analysis process. Therefore, it is impossible to compare the size between amorphous MnO_2 and crystalline MnO_2. Generally, the amorphous MnO_2 has a smaller size than crystalline MnO_2 (Figure S2).

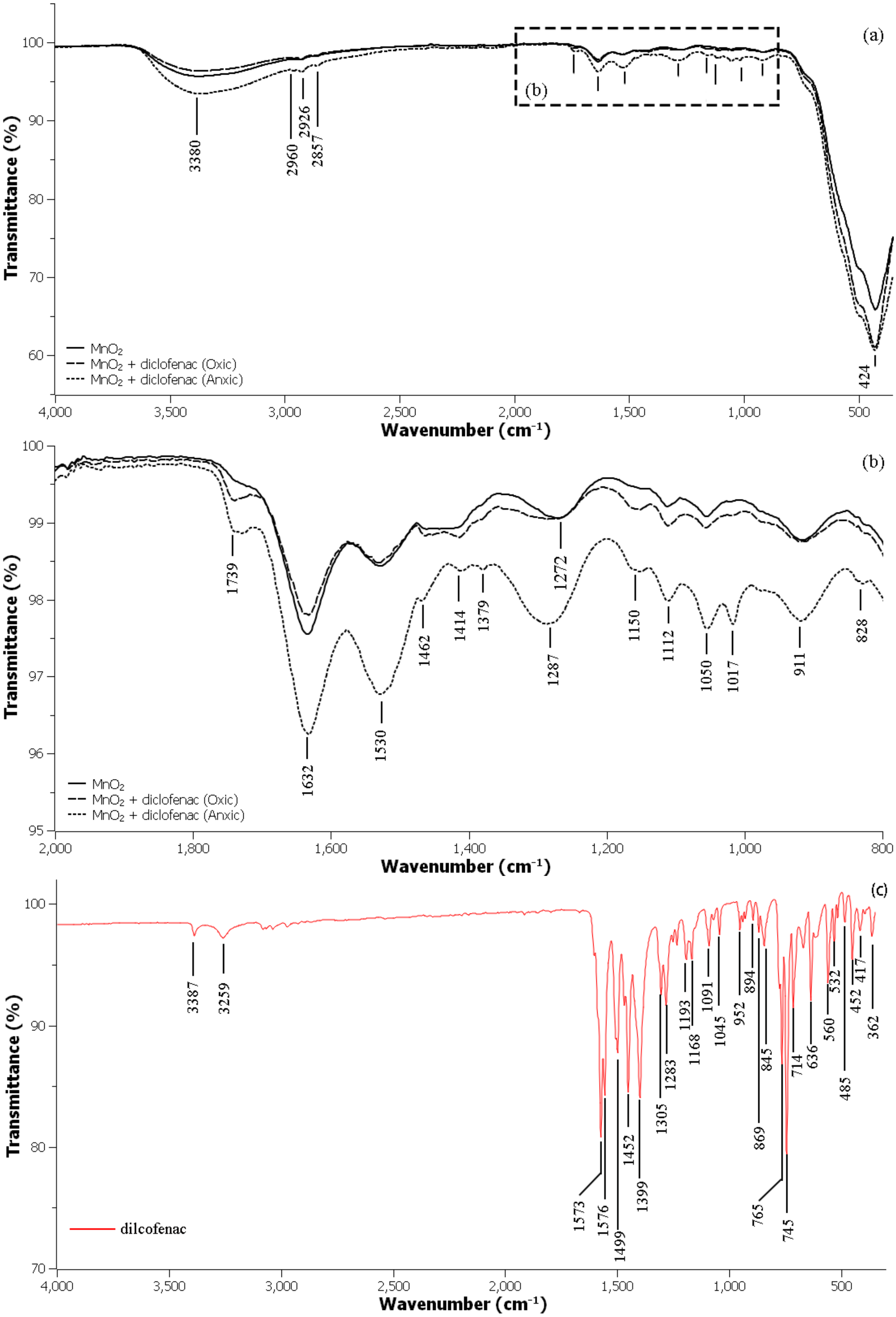
(a)
(b)
Transmittance (%)
Wavenumber (cm⁻¹)
3380
2960
2926
2857
424
MnO2
MnO2 + diclofenac (Oxic)
MnO2 + diclofenac (Anxic)
1739
1632
1530
1462
1414
1379
1287
1272
1150
1112
1050
1017
911
828
(c)
3387
3259
1573
1576
1499
1452
1399
1305
1283
1193
1168
1091
1045
952
894
869
845
765
745
714
636
560
532
485
452
417
362
dilcofenac

FIG. S6. FTIR spectra of MnO_2 before (solid line) and after reacting with diclofenac under oxic condition (dash line) and anoxic condition (dot line) at (a) 350 ~ 4000 cm^{-1}; and (b) 800 ~ 2000 cm^{-1}. The pure diclofenac (c) was used as a reference.

The bare MnO_2 under oxic and anoxic conditions are the same, therefore, we only use the FTIR spectra of oxic MnO2 in Figure S6.The FTIR spectra show the MnO_2 changed before and after reacting with diclofenac. The spectra of bare MnO_2 is similar to previous study [2]. The broad peak at 3380 cm^{-1} was assigned to the stretching vibration of -OH group in water and Mn-O-H. The peaks at 1634 cm^{-1} and 1056 cm^{-1} were assigned to the bending vibrations of the -OH in Mn-OH. These peaks are also the indications of the reactive sites on MnO_2 surface. The peak at 428 cm^{-1} was assigned to the bending vibration of Mn-O [2, 3].

Due to the extremely huge amount of MnO_2 added into the system comparing to diclofenac, the spectra of MnO_2 after reacting are still similar to the bare MnO_2. However, there are still some peaks appeared between 800 ~ 2000 cm^{-1} showing the MnO_2 is changed. Three new peaks at 1379 cm^{-1} , 1017 cm^{-1} and 828 cm^{-1} were not attributed to the pure diclofenac either (Figure S6 (c)). Therefore, they are probably from the intermediates. The peak at 1379 cm^{-1} is assigned to alkynes, aromatics or C=O, the peak at 1017 cm-1 is assigned to C-O (2-bands) and the peak at 828 cm^{-1} is assigned to N-H wagging [3].

In addition, the peak shift and peak change were also observed. The peak at 1632 cm^{-1} decreased after reacting with diclofenac under oxic condition while it increased under anoxic conditions. The higher peak probably indicates that the reactive sites are activated and more available for diclofenac. Another peak shift is observed from 1272 cm^{-1} to 1287 cm^{-1}. The peak also larger. That peak is assigned to alkynes or NO_2.

All these peaks showed that under anoxic conditions, the MnO_2 changed more than that under oxic conditions. The change may be caused by the intermediates. The change of MnO_2 under anoxic conditions can be the reason why anoxic conditions promote diclofenac removal.

Reference

[1] Y. He, N.B. Sutton, H.H.H. Rijnaarts, A.A.M. Langenhoff, Degradation of pharmaceuticals in wastewater using immobilized TiO2 photocatalysis under simulated solar irradiation, Applied Catalysis B: Environmental 182 (2016) 132-141.

[2] G. Chen, L. Zhao, Y.-h. Dong, Oxidative degradation kinetics and products of chlortetracycline by manganese dioxide, Journal of Hazardous Materials 193 (2011) 128-138.

[3] R.M. Silverstein, F.X. Webster, D.J. Kiemle, D.L. Bryce, Spectrometric Identification of Organic Compounds, Wiley2014.
